# Full genetic characterization and epidemiology of a novel amdoparvovirus in striped skunk (*Mephitis mephitis*)

**DOI:** 10.1038/emi.2017.13

**Published:** 2017-05-10

**Authors:** Marta Canuti, Hillary E Doyle, Ann P Britton, Andrew S Lang

**Affiliations:** 1 Department of Biology, Memorial University of Newfoundland, St John’s, NL A1B3X9, Canada; 2 Animal Health Centre, BC Ministry of Agriculture, Abbotsford, BC V3G2M3, Canada

**Keywords:** Aleutian disease, Aleutian mink disease virus, amdoparvovirus, host range, mink, parvovirus, skunk, viral recombination

## Abstract

*Amdoparvovirus* is a newly defined parvoviral genus that contains four species (*Carnivore amdoparvovirus 1–4*), including the well-known Aleutian mink disease virus (AMDV). Amdoparvoviruses cause an immune-associated and often lethal wasting syndrome in Mustelidae and Caninae hosts. In this study, we molecularly investigated amdoparvoviruses detected in 44 striped skunks (*Mephitis mephitis*) found dead in and around Vancouver, British Columbia, Canada. Some of the animals exhibited pathological changes compatible with amdoparvovirus-associated disease. The nearly complete genomic sequence was obtained for seven different strains and our analyses show how this virus, which we named skunk amdoparvovirus (SKAV), should be classified as a separate species within the genus (proposed *Carnivore amdoparvovirus 5*). We detected co-infections, recombinant genomes, at least three separate viral lineages, and preliminary evidence for geographic segregation of lineages. Furthermore, we proved that similar viruses, only partially characterized in previous studies and labeled as AMDV, circulate in skunks from other distant areas of North America (Ontario and California) and found evidence for spillover events in mink (*Neovison vison*). Although SKAVs are capable of causing disease in infected animals, a high proportion of sub-clinical infections has been observed, suggesting these animals might act as asymptomatic carriers and pose a threat to wild and captive carnivores. Finally, we highlight the need for more specific diagnostic tests and further molecular investigations to clarify the epidemiology and host- and geographical distributions of amdoparvoviruses in terrestrial carnivores, especially because the whole spectrum of viral diversity in this group is likely still unknown.

## INTRODUCTION

The genus *Amdoparvovirus*, within the family *Parvoviridae* and subfamily *Parvovirinae*, was recently defined by the International Committee on Taxonomy of Viruses (ICTV)^1^ after the discovery of several novel parvoviruses sharing genetic and virological characteristics with the Aleutian mink disease virus (AMDV) between 2011 and 2014.^2^ In addition to AMDV, classified as *Carnivore amdoparvovirus 1*, whose primary hosts are members of the Mustelidae family and possibly other furbearing animals, this genus includes a variety of viruses found in the Caninae. These are the gray fox amdoparvovirus (GFAV) or *Carnivore amdoparvovirus 2*^3^ and the proposed *Carnivore amdoparvovirus 3* or raccoon dog and fox amdoparvovirus (RFAV)^4^ and *Carnivore amdoparvovirus 4* or red fox fecal amdovirus (RFFAV).^5^ These small single-stranded DNA viruses possess a genome of ~4.8 kb that contains two major open reading frames (ORFs).^2^ These ORFs, one encoding three non-structural proteins (NS1, NS2 and NS3) and the other two structural proteins (VP1 and VP2), are characterized by different evolutionary dynamics.^6^

Amdoparvoviruses are known to cause an immune complex-mediated wasting syndrome that is often fatal, especially in highly susceptible hosts, and are responsible for the occurrence of vast epidemics in farms, frequently associated with great economic losses.^2, 4, 6^ Farm-derived strains also represent a threat for wild animal populations as escape of animals can result in the introduction of viruses to new geographic areas, where novel host species can get infected.^2, 7, 8^ Furthermore, infected animals do not always develop clinical signs and healthy carriers might have a significant role in the diffusion and distribution of viruses in the wild and to farms by acting as reservoir hosts.^9, 10, 11^ As disease severity seems to be influenced by both host and viral genetic factors,^2^ the study of amdoparvoviral infections in several different hosts is essential to achieve a full understanding of the transmission dynamics and pathogenicity potential of these viruses.

Amdoparvoviruses have been found to be highly prevalent in skunks and, despite pathological examinations confirming lesions compatible with amdoparvoviral infection in several cases,^10, 12, 13, 14^ the proportion of sub-clinical infections seems to be high, suggesting that infections in these animals are primarily asymptomatic.^10, 15^ Despite their potentially fundamental role in amdoparvoviral epidemiology, only a few studies have looked at the genetic makeup of AMDV-like viruses in these animals ^11, 13^ and, therefore, their genetic diversity and molecular features are largely unknown. In this study we fully molecularly characterized amdoparvoviruses detected in mainly asymptomatic striped skunks (*Mephitis mephitis*) from in and around Vancouver, BC, Canada, and we studied their molecular epidemiology and distribution in North America. Furthermore, our sequence and phylogenetic analyses showed that this virus, which we have named skunk amdoparvovirus (SKAV), is divergent from all known members of the genus *Amdoparvovirus* and we evaluated its potential classification as a novel amdoparvoviral species (proposed *Carnivore amdoparvovirus 5*).

## MATERIALS AND METHODS

### Virus amplification and sequencing

**Figure 1:**
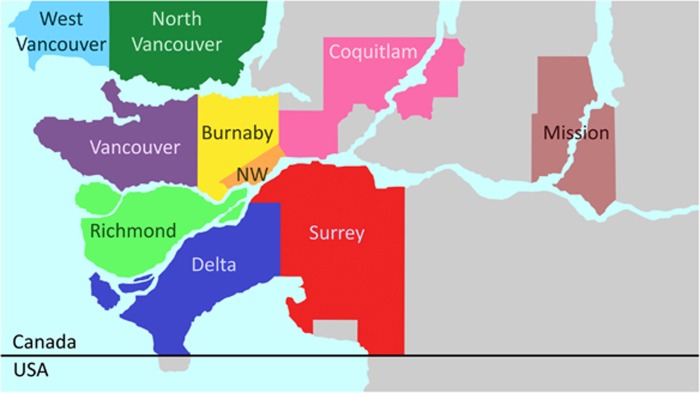
Areas in British Columbia corresponding to sample collection sites. New Westminster, NW.

We analyzed viral DNA isolated from 44 striped skunks found dead or injured severely in and around the Vancouver area (Figure 1), BC, Canada, between March 2011 and May 2015, which were previously identified as amdoparvovirus-positive.^10^ The causes of death of these animals were various (mainly traumas and not related to infectious diseases) and only 4.6% showed pathological evidence of Aleutian disease. A complete description of the post-mortem examinations for this population is available in Britton *et al.*^10^ The nearly complete genomic sequences of seven viruses and the complete NS1 ORF of one additional virus were obtained as previously described.^6^ The primers AMDO_3F (5′-GGA TGG TTA CTW TGC TGC TG-3′) and AMDO_2R (5′-ACA TKC CTG GTG TTA YTT TRG-3′) were used to amplify and sequence a 998-nt long fragment of the NS1 region (corresponding to nt 892–1889 of the AMDV-G reference sequence, accession number JN040434) for the molecular epidemiological investigation. For viruses showing evidence of multiple infections or intra-host polymorphisms, amplified fragments were cloned into a plasmid vector before sequencing.^6^

Ten spleen samples were also obtained from AMDV-positive mink from five different farms throughout British Columbia for further comparisons. DNA was extracted from mink spleen tissues using the DNeasy Blood and Tissue kit (Qiagen, Toronto, ON, Canada) and the complete NS1 ORF was sequenced as previously described.^6^

### Phylogenetic and sequence analyses

Sequences obtained in this study were compared to several AMDV, RFAV, GFAV and RFFAV sequences downloaded from GeneBank (see Supplementary Table S1 for accession numbers and details). Splicing sites were determined following what was experimentally demonstrated for AMDV,^16^ donor and acceptor sites were confirmed using NNSPLICE,^17^ and splicing events were reproduced *in silico* to determine the complete coding sequences for all viral proteins, which were then translated into amino acid sequences.

Nucleotide and protein sequences were aligned with ClustalX 2.1^18^ and alignments were manually edited when necessary. A model test to identify the best model for distance estimation was performed for each alignment and maximum-likelihood trees^19^ were constructed using MEGA 7.0.18.^20^ Bootstrap tests^21^ with 1000 replicates were performed to test the robustness of the analyses and only clusters supported by bootstrap values >70% were considered valid. Average identities (1—p-distances) for sequence pairs within and between groups were calculated with MEGA (with pairwise gap removal).

### Recombination analyses

Alignments of complete genomes were analyzed for the presence of recombinant strains using the RDP 4.55 software package^22^ and only events supported by at least three methods (*P*<0.05) were accepted for confirmation. All amdoparvoviruses for which a complete genomic sequence is known were included in the analysis but viruses other than SKAV were used only for tree topology estimates. Potential recombination events were evaluated with SimPlot software 3.5.1^23^ (window: 500 bp; step: 60 bp; gap strip: on; model: F84; method: maximum-likelihood; bootstrap replicates: 1000) and novel phylogenetic trees were built with sub-portions of the alignment included between putative breakpoints.

### Accession numbers

All sequences obtained in this study have been deposited in GenBank under accession numbers KX981972–KX981981 for partial AMDVs, KX981927–KX981971 for partial SKAVs and KX981920–KX981926 for the nearly complete SKAV genomes.

## RESULTS

### *Carnivore amdoparvovirus 5*, a proposed new viral species within the genus *Amdoparvovirus*

We obtained the nearly complete genomic sequences of seven SKAVs that were identified in mainly asymptomatic striped skunks from the Vancouver area (two from Vancouver, one from each of North Vancouver, New Westminster, Surrey and Richmond, Figure 1; and one was found dead on a mink farm at an undisclosed location in Southern British Columbia). We also obtained the complete NS1 ORF sequence from an additional SKAV (sample collected in Mission). The nearly complete genomic sequences include the entire protein-coding regions but lack the untranslated termini, which in parvoviruses form hairpin structures that are particularly difficult to amplify.

The genomic organization was consistent with those of other parvoviruses and protein sizes, as predicted by *in silico* splicing, were conserved across all genomes and for all SKAV proteins (NS1: 641 aa, NS2: 114 aa, NS3: 71 aa, VP1: 677 aa, VP2: 634 aa; details provided in Supplementary Table S2). At the C-terminal side of NS1 we identified the four helicase motifs (aa 427–522) present in all parvoviruses,^24^ which showed high conservation among all amdoparvoviruses (Supplementary Figure S1). In particular, Walker motif A was identical in all amdoparvoviruses, motif C of GFAV was different at one position from all other viruses and motif B’ differed by one or two positions between SKAVs and the other viruses. Finally, the last hydrophobic residue of motif B was substituted by a neutral aa in some AMDVs and SKAVs. As expected, the phospholipase A2 motif was absent from the VP1 of SKAVs, as with all other amdoparvoviruses.^24^ Finally, in contrast to AMDV and RFAV, we observed no variation in length of the glycine stretch at the beginning of VP2.^6^

**Figure 2:**
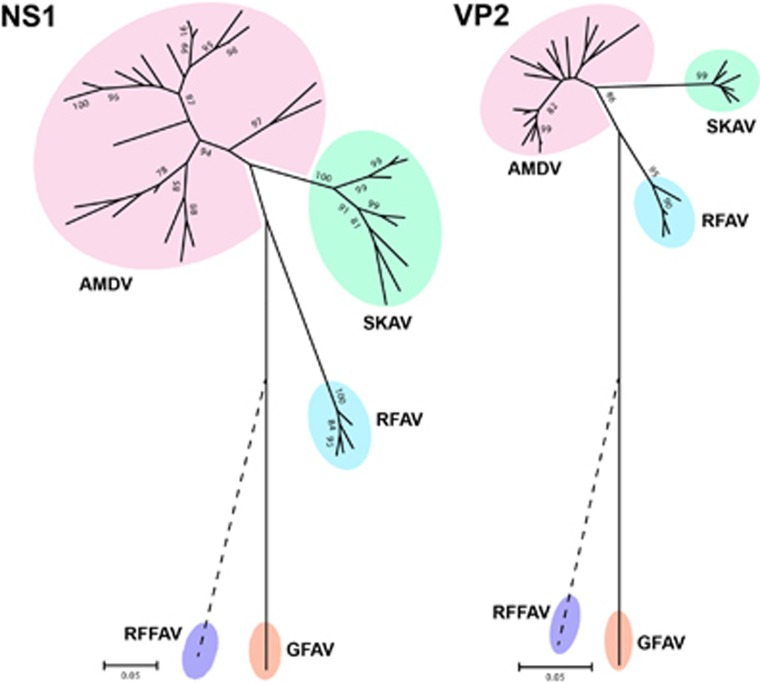
Phylogenetic analyses of the genus *Amdoparvovirus*. Trees were built with amino acid sequences of the complete NS1 and VP2 proteins using the maximum-likelihood method^19^ based on the JTT^25^ (NS1) and rtREV^26^ (VP2) plus Gamma models. Viral species: Aleutian mink disease virus (AMDV, *Carnivore amdoparvovirus 1*), gray fox amdoparvovirus (GFAV, *Carnivore amdoparvovirus 2*), racoon dog and fox amdoparvovirus (RFAV, proposed *Carnivore amdoparvovirus 3*), red fox fecal amdovirus (RFFAV, proposed *Carnivore amdoparvovirus 4*) and skunk amdoparvovirus (SKAV, proposed *Carnivore amdoparvovirus 5*). The phylogenetic placement of RFFAV was manually indicated on the trees (dotted line) based on previous analyses.^2^

Two distinct phylogenetic trees were built using the complete NS1 and VP2 protein sequences (Figure 2) that included representative members of almost all amdoparvoviral clades identified in skunks, mink, foxes and raccoon dogs (see Supplementary Table S1 for details). The exception was RFFAV, whose genomic sequence is not complete, and it was therefore manually placed on the trees (dotted line) based on previous analyses.^2^ In both analyses SKAVs clustered separately from other amdoparvoviral species and formed an independent lineage located between AMDV and RFAV. All viruses identified in British Columbia farmed mink (BCM), including those originating from the farm where one of the skunks under investigation was found, clustered within the AMDV clade (see Supplementary Figure S2 for phylogenetic trees *in extenso*).

Finally, pairwise sequence identities within and between each group were calculated for both proteins (Table 1). Overall, the mean sequence identities between groups were <85% (range: 59.1%–80.4%) for NS1 and <95% (range: 72.6%–91.2%) for VP2. In particular, SKAVs were the most similar to AMDVs (average identities: 80.4% for NS1 and 91.2% for VP2). The ICTV rules for classification of viruses within the family *Parvoviridae* state that the NS1 sequences of viruses from one species must ‘show >85% amino acid sequence identity, while diverging by >15% from viruses in other species’,^27^ and we therefore concluded that SKAVs could be classified as a separate viral species, tentatively named *Carnivore amdoparvovirus 5*.

### Identification of recombinant viruses

**Figure 3:**
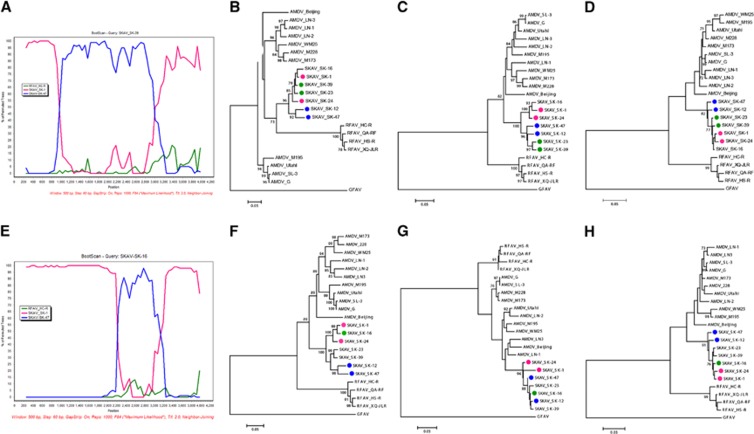
Recombination analyses of SKAVs. The BootScan analysis is displayed for recombinant sequences SK-39 (**A**) and SK-16 (**E**). Trees built with partitions of the alignment between breakpoints are illustrated in **B** (nt 69–1030 of the SKAV_SK-1 complete genome), (**C**) (nt 1031–3192) and (**D**) (nt 3193–4225) for the first recombination event and (**F**) (nt 69–2332), (**G**) (nt 2333–2993) and (**H**) (nt 2994–4225) for the second. The evolutionary histories were inferred with the maximum-likelihood method^19^ based on the GTR (**B**, **C** and **F**), HKY (**D** and **G**) and TN93 (**H**) plus gamma models.^29, 30, 31^ Putative recombinant sequences are indicated by green dots, the possible minor parents by blue dots and the possible major parents by pink dots.

Recombination has frequently been observed in parvoviruses,^28^ including AMDV,^6^ and we therefore investigated whether chimeric genomes were present among the fully sequenced SKAVs. We found evidence for at least two recombination events and identified two sequences with the same chimeric pattern (Figure 3).

The first event involved two potential chimeric sequences (SK-23 and SK-39) and two putative breakpoints, located at approximately nt 1000 and 3200 of the SK-1 genomic sequence. The second event involved one chimeric sequence (SK-16) and the two putative breakpoints were located at approximately nt 2300 and 3000 of the SK-1 genomic sequence. Both events could be clearly visualized by the BootScan analysis performed with Simplot (Figures 3A and 3E), where the differential clustering of different portions of the genome with the minor and major parental strains were highly supported.

Finally, phylogenetic trees were built with sub-portions of the alignment that included the regions between putative breakpoints (Figures 3B–3D and 3F–3H). As expected, the chimeric sequences were located in different clades in the separate trees, confirming the occurrence of past recombination. The first breakpoint was well supported and associated with high bootstrap values of the different clades in trees built with sub-genomic regions before and after the breakpoint (Figures 3B and 3C). However, phylogenetic relationships among strains were more difficult to resolve with partial sequences of the 3’-half of the genome (Figures 3D, 3G and 3H), possibly because of high sequence identity in this area, and the confidence for the clustering in these trees was poor. This analysis should therefore be repeated once more SKAV sequences become available.

### Molecular epidemiology of SKAVs in British Columbia

**Figure 4:**
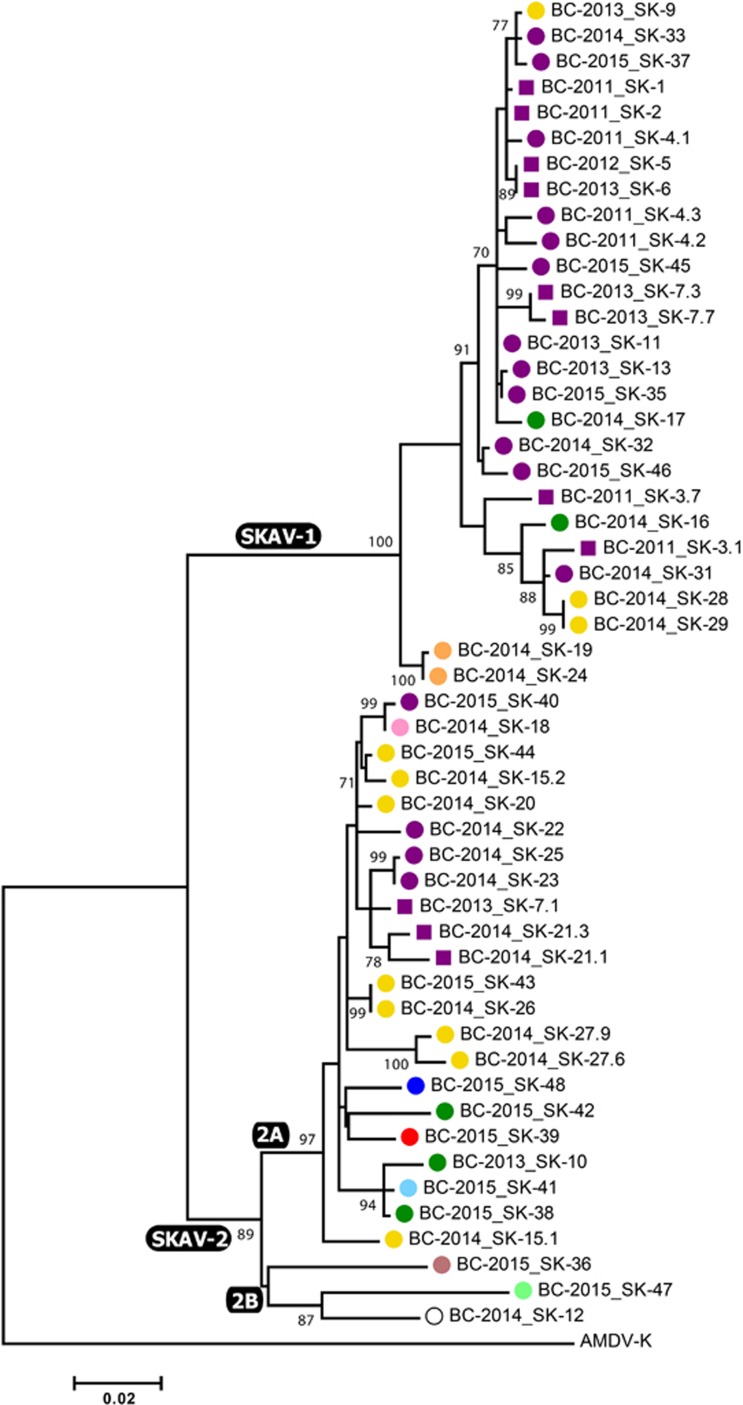
Phylogenetic analysis of partial NS1 nucleotide sequences of SKAVs identified in striped skunks from in and around Vancouver. The evolutionary history of the region included between nucleotides 706–1662 of SK-1 complete genome was inferred using the maximum-likelihood method^19^ based on the HKY-85^30^ plus Gamma plus I model. Each virus is indicated by a different colored shape that corresponds to sample collection sites as illustrated in Figure 1 (purple squares indicate Stanley Park in Vancouver). The empty circle indicates a skunk that was found dead on a mink farm. The name of each strain is preceded by the province (British Columbia) and year of collection. Lineages and sub-lineages are labeled at the corresponding nodes.

In total, we analyzed 44 samples collected from dead skunks found in and around Vancouver, including one animal (SK-12) that was found dead on a mink farm (cause of death was cellulitis)^10^ from which we also obtained AMDV sequences from mink (BCM-1 and BCM-3). The chromatograms obtained from several samples showed the presence of double peaks at >1 site, potentially indicative of multiple infections, and such amplicons were subjected to cloning before sequencing. Overall, this resulted in 52 SKAV nucleotide sequences considered in our molecular epidemiological investigation. In most cases, clones obtained from the same animal differed from each other at only a few positions and belonged to the same phylogenetic group, making the distinction between intra-host mutation and co-infection impossible. The presence of multiple distinct viruses simultaneously infecting one animal could be confirmed in only one case (SK-7) as viruses from two different lineages (see below) could be identified in this animal (Figure 4).

The phylogenetic tree built with an ~950-nt region of the NS1 ORF (Figure 4) revealed the existence of two distinct lineages, which we named SKAV-1 and SKAV-2 and that included viruses identified in 21 (47.7%) and 24 (54.5%) animals, respectively. A very high viral diversity was observed (overall mean pairwise identity: 94.5%). The average pairwise identity between sequences belonging to the two lineages was 91.4% (range: 89.6%–93.3%) and a higher diversity was observed for SKAV-2 (within lineage identity: 97%, range: 92.5%–100%) compared to SKAV-1 (within lineage identity: 98.2%, range: 95.9%–100%). In fact, SKAV-2 could be further dived into separate sub-lineages: SKAV-2A (average within sub-lineage identity: 97.9%, range: 95.6%–100%), which included the vast majority of the SKAV-2 sequences (*n*=22), and SKAV-2B, which contained three viral sequences sharing lower sequence identities with each other (average: 93.9%, range: 93.4%–94.6%) and that could potentially represent three separate sub-lineages. The complete set of sequence identities within and between clades and sub-clades is available in the Supplementary Table S3.

No clear geographical distribution of strains was observed. However, the SKAV-2 lineage was more diverse in terms of geographic origin as it included viruses obtained from each sampled location, including the only two viruses sampled south of the Fraser River. Interestingly, sub-clade 2B contained viruses sampled in the most isolated areas: Lulu Island (where Richmond is located), Mission (~40 km from the next nearest sampling location) and the mink farm (south of the Fraser River, ~30 km from Mission and at least 60 km from Richmond). Finally, no temporal pattern was observed in the data, although no viruses from 2011 and 2012 were observed within the SKAV-2 lineage.

### Host-distribution and diversity of SKAVs in North America

A thorough search of previous literature and in the NCBI database, which considered every amdoparvoviral sequence ever deposited in GenBank or reported in publications about AMDV-like viruses in skunks, revealed that similar viruses are also circulating in other locations in North America but distant from British Columbia (Western Canada). Specifically, these were identified in Ontario (Eastern Canada) and California (Southwestern USA) and corresponded to one Californian and 15 Canadian striped skunks.^11, 13^ Interestingly, evidence of SKAV infections was also found in one likely escaped domestic mink^32^ and another mink of unknown origin^6^ in Ontario. However, all of these viruses were not completely characterized in the previous studies and were therefore not recognized as a separate species and were labeled as AMDV.

**Figure 5:**
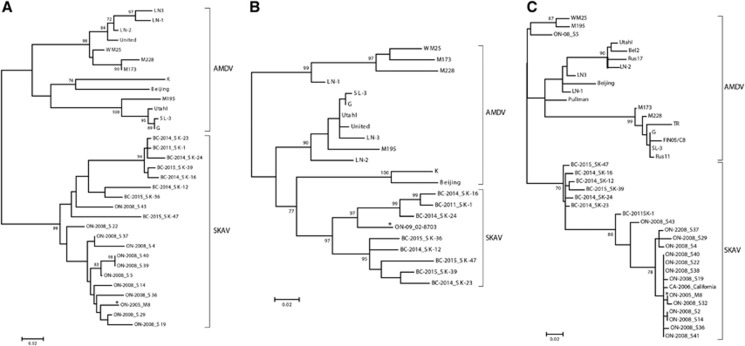
Phylogenetic trees of AMDVs and SKAVs constructed with partial nucleotide sequences of the NS1 (**A** and **B**) and VP2 (**C**) regions. Trees have been constructed with alignments based on regions between nucleotides 602–922 (**A**), 1229–1672 (**B**), and 3018–3227 (**C**) of the AMDV-G complete genome (accession number JN040434). The evolutionary histories were inferred using the maximum-likelihood method^19^ based on the HKY^30^ (NS1) and TN93^31^ (VP2) models. AMDV and SKAV species are indicated by square parenthesis and the name of each strain is preceded by the location (British Columbia, BC; Ontario, ON; California, CA) and year of collection. SKAV sequences identified in mink are labeled with an asterisk.

We studied the relationships among SKAVs identified in different locations and in different hosts by performing additional phylogenetic analyses. Unfortunately, only partial sequences of different portions of the genome were available for the viruses from California and Ontario. Therefore, we were not able to compare all viruses together and three separate phylogenetic trees were required to properly compare the various sequences (Figure 5). Sequences from both Ontario and California, for both NS1 and VP2 partial ORFs, clustered separately from AMDV and together with the SKAVs identified in British Columbia. Only two out of the 18 considered strains (the skunk virus S43 and the mink virus ON09-02-8703) were close to the British Columbia viruses, and the other strains were in an independent clade, indicating the existence of at least a third SKAV lineage. Overall, SKAVs shared ~93% sequence identity within the two NS1 regions considered (93% and 92.7%, respectively; range: 88.2%–100%) and 94.4% (range: 88.1%–100%) within the hypervariable region of VP2, and the strains identified in mink were included within the clades containing the sequences from skunks.

## DISCUSSION

Amdoparvoviruses cause persistent infections in their hosts that can lead to a serious wasting syndrome, also known as Aleutian disease, associated with hypergammaglobulinemia, kidney failure and often death. AMDV, the first-identified and most-studied member of the group, is also known to cause an acute respiratory infection in mink kits, which can progress into fulminant pneumonia, and has been associated with complications during pregnancy.^2^ The virus we describe in this study is likely responsible for a similar disease in striped skunks as a proportion of the studied infected animals showed pathological evidence of Aleutian disease.^10, 13^ This virus, which we named Skunk amdoparvovirus (SKAV) as this is the only amdoparvovirus identified so far capable of establishing a clinically evident disease in skunks, is divergent enough from all the other members of the genus *Amdoparvovirus* to be classified as a separate species. According to the most recent ICTV classification rules,^27^ and also taking into account the latest published discoveries,^4, 5^ we propose the taxonomic species designation of *Carnivore amdoparvovirus 5* for SKAV.

Amdoparvoviruses are principally studied because they cause vast epidemics in farms, often associated with great economic losses,^6, 33^ which are strikingly difficult to overcome and prevent as there are no available cures or treatments and because the great environmental stability of the viral particles makes them difficult to eradicate.^2^ However, because of the wide range of potentially susceptible hosts, these viruses have also raised major concerns outside of the fur industry as they represent a serious risk for wild animals and pose a threat to endangered species.^7, 8^ The presence of asymptomatic individuals, whose existence has already been proven among mink and ferrets,^34, 35^ certainly facilitates viral dispersion, making new hosts more accessible. The vast majority of the skunks that were sources or viruses for this study presented with an apparently sub-clinical infection,^10^ consistent with a presumed role of these animals as amdoparvovirus carriers and natural reservoir hosts, as already postulated by others.^11^ However, it is also possible that in some cases the infection was detected before signs of disease became evident, as the most common cause of death/killing in these animals was trauma, and further studies are required to clarify the full spectrum of clinical signs associated with SKAV infection. We have also identified two cases of SKAV-infected mink, demonstrating the capability for SKAVs to cross species barriers and infect other hosts. Furthermore, one of the skunks under investigation was found dead within the borders of a mink farm, illustrating how close contacts between skunks and mink are possible and that skunks can carry these viruses into farms, where close contacts between animals facilitate the establishment of co-infections and lead to recombination.^6^ Interspecies recombination has been already documented among members of the family *Parvoviridae*^28, 36^ and farmed mink might represent a potential mixing vessel for AMDV and SKAV. It is currently unknown whether SKAVs can infect other carnivore species other than mink and future molecular investigations will be required to answer this question.

As already observed for AMDV,^6, 32, 37^ a high diversity was observed among SKAVs, with recombinant genomes and at least three separate viral lineages detected. Nevertheless, we acknowledge that the whole spectrum of genetic variability of SKAVs is likely still largely unknown and hypothesize that additional diversity and lineages remain to be discovered. This is primarily based on the observation that highly divergent viruses forming single-strain clades were identified, and because there have been only a few studies investigating genetic characteristics of amdoparvoviruses in skunks. Furthermore, as we already postulated elsewhere,^2, 6^ it is reasonable to believe that the whole spectrum of amdoparvoviruses infecting terrestrial carnivores is currently unknown and we reemphasize here the necessity of performing more molecular epidemiological investigations in these animals to expand our current knowledge of diversity within this viral group.

On the basis of our epidemiological investigation, these viruses are circulating in at least three distinct geographical regions covering distal regions of continental Canada (British Columbia and Ontario) and in the southern USA (California) and, according to available epidemiological data, these viruses are highly prevalent (detected prevalence of 86% in British Columbia and 32% in Ontario).^10, 11^ Although the genetic similarity of viruses detected in these three areas is high, it appears that distinct lineages might be circulating in different locations. However, the available sequence data from Ontario and California were insufficient to perform a detailed comparison and, therefore, further sequencing efforts should be carried out to complete this investigation. At a more local level, when examining viruses obtained from Vancouver and surrounding areas, we have also found evidence for geographic effects on the relationships among strains. Viruses obtained from Lulu Island, Mission and the mink farm (the last two of which are ~40 and 60 km from the next closest sample collection locations, respectively) were genetically different from what was identified in Vancouver-proper and the other surrounding districts. Furthermore, our results suggest that water could act as physical barrier for viral spread as a different distribution of strains was observed in areas north and south of the Fraser River and within Lulu island. However, the number of analyzed samples was very small and a more intense and prolonged sampling within these areas is needed to better resolve the epidemiological dynamics, transmission behavior, and phylogeographic pattern of SKAVs.

Even though SKAV and AMDV are sufficiently genetically distant to be considered separate species, they still share a high genetic similarity and this is especially pronounced within the gene encoding the capsid protein, which is known to be more conserved among amdoparvoviruses.^2, 6^ Because of this, primers normally utilized for the molecular diagnosis of AMDV could be used to amplify SKAVs^10, 11^ and the two viruses could be confused without a more thorough genetic characterization. In addition, this high genetic similarity most likely translates into an elevated antigenic similarity, which might result in cross-reactivity between antibodies and antigens of different viruses and result in misdiagnosis when immunological assays are employed. It is therefore possible that previous studies that reported evidence for AMDV in skunks actually detected other amdoparvoviral species or antibodies against them and, therefore, viral sequencing has to be included in future investigations for correct data interpretation. Two previous studies have analyzed the genetic characteristics of amdoparvoviruses in skunks,^11, 13^ and the detected viruses can be classified as SKAVs in both cases. Indeed, to our knowledge, SKAV is the only amdoparvovirus ever identified in skunks and more studies are necessary to evaluate whether AMDV is actually capable of infecting striped skunks and if other amdoparvoviral species can be found in these animals. Obtaining sequence data from suspected cases of Aleutian disease in skunks, including those already published if possible,^12, 14, 15^ therefore has to be considered a priority, together with the development of more specific diagnostic tests.

In summary, we describe here a novel amdoparvovirus that infects striped skunks (skunk amdoparvovirus or SKAV), which is highly prevalent in Canada and appears to be widespread in North America. Infected animals show signs of disease typically associated with other amdoparvoviruses but a high proportion of sub-clinical infections has been observed, suggesting asymptomatic carriers could be common. These animals might be responsible for an efficient distribution of viruses in the wild and transmit the infection to other animal species, and this hypothesis is supported by the observed spillover events in mink. As this virus might pose a threat to wild and captive carnivore populations and the entire genetic spectrum of circulating lineages is unknown, we strongly highlight the need to perform more molecular epidemiological investigations to study the distribution and diversity of amdoparvoviruses in skunks.

## Figures and Tables

**Table 1:** Average (range) pairwise percentage sequence identities (1—p-distance) within and between different amdoparvoviral species for NS1 (bottom) and VP2 (top and in italics) proteins

	**AMDV**	**SKAV**	**RFAV**	**GFAV**	**RFFAV**^b^
**AMDV**	*95.7 (92.4–99.2)*	*91.2 (89.7–93.1)*	*91.2 (89.3–92.7)*	*79.3 (78.4–79.8)*	*75.0 (73.8–76.4)*
	88.3 (81.6–99.8)				
**SKAV**		*98.2 (97.4–99.5)*	*88.6 (87.8–89.9)*	*78.7 (78.2–79.1)*	*72.6 (72.0–73.8)*
	80.4 (77.5–83.0)	90.1 (85.6–97.3)			
**RFAV**			*97.2 (95.8–98.3)*	*80.0 (79.5–80.4)*	*75.6 (75.4–76.5)*
	74.3 (71.9–76.4)	73.5 (72.5–75.3)	95.1 (94.4–96.4)		
**GFAV**				NA^a^	*80.8*
	67.6 (66.1–70.0)	67.0 (66.0–68.0)	65.0 (64.3–65.8)	NA^a^	
**RFFAV**^b^					NA^a^
	60.4 (57.7–62.4)	61.4 (59.0–64.0)	59.1 (58.2–59.8)	63.5	NA^a^

Abbreviations: Aleutian mink disease virus, AMDV; skunk amdoparvovirus, SKAV; raccoon dog and fox amdoparvovirus, RFAV; gray fox amdoparvovirus, GFAV; red fox fecal amdovirus, RFFAV.

a NA: not available as only one member is known for these species.

b Based only on partial sequences (189 aa for NS1 and 276 aa for VP2) as the complete genomic sequence of RFFAV is not available.
