# Supplementary material for: Full genetic characterization and epidemiology of a novel amdoparvovirus in striped skunk (Mephitis mephitis)
Source: Emerg Microbes Infect. 2017 May 10;6(5):e30–. doi: 10.1038/emi.2017.13 (PMC5520478; doi:10.1038/emi.2017.13)
Supplement: Supplementary Figure S1 [file emi201713x1.pdf]

**Supplementary Figure S1. Helicase domains in members of the genus *Amdoparvovirus***
A sequence logo for each species was generated with Weblogo 3. Walker domains are indicated in square boxes.

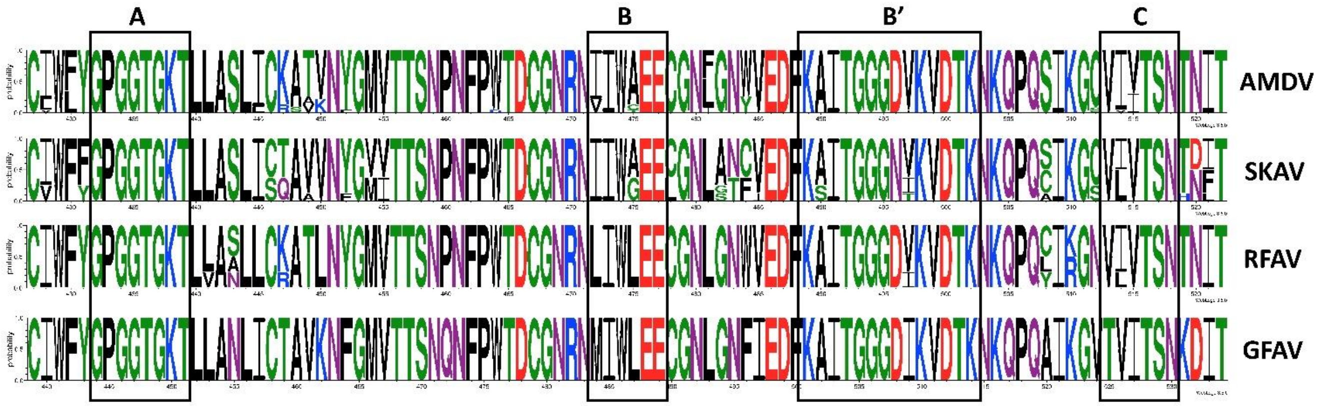


Figure S1
