# Supplementary material for: Full genetic characterization and epidemiology of a novel amdoparvovirus in striped skunk (Mephitis mephitis)
Source: Emerg Microbes Infect. 2017 May 10;6(5):e30–. doi: 10.1038/emi.2017.13 (PMC5520478; doi:10.1038/emi.2017.13)
Supplement: Supplementary Figure S2 [file emi201713x2.pdf]

Supplementary Figure S2. Phylogenetic tree of members of the genus *Amdoparvovirus* constructed with amino acid sequences of the full NS1 (A) and VP2 (B) proteins

The evolutionary histories were inferred using the maximum likelihood method based on the JTT model for NS1 and rtREV model for VP2, identified as the best fitting models after the model test analysis using MEGA 7. A discrete Gamma distribution was used to model evolutionary rate differences among sites. The outcome of the bootstrap analysis is shown next to the nodes, and branch lengths are proportional to genetic distances as indicated by the scale bar. Each viral species is indicated by square parenthesis.

A

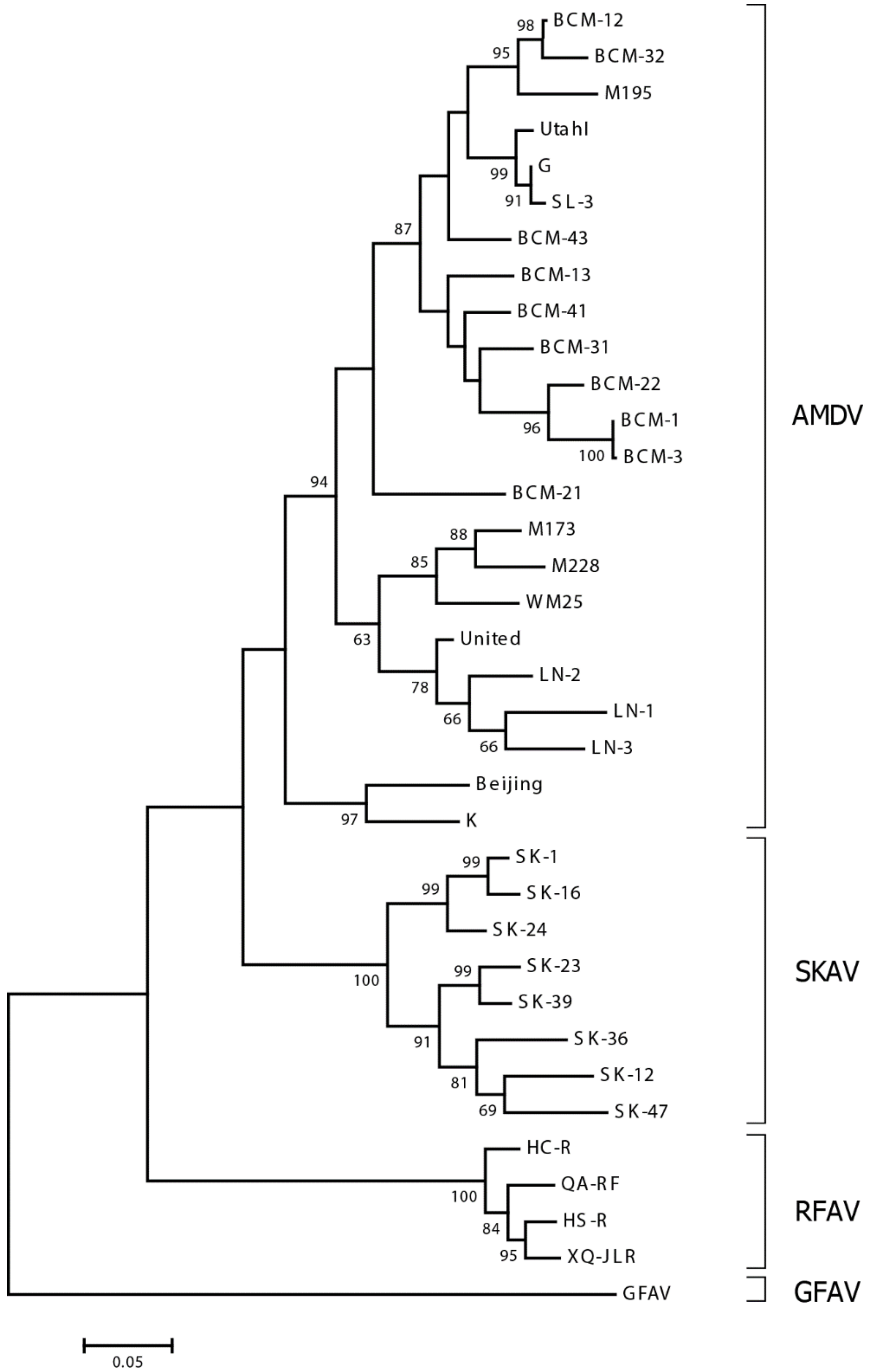

B

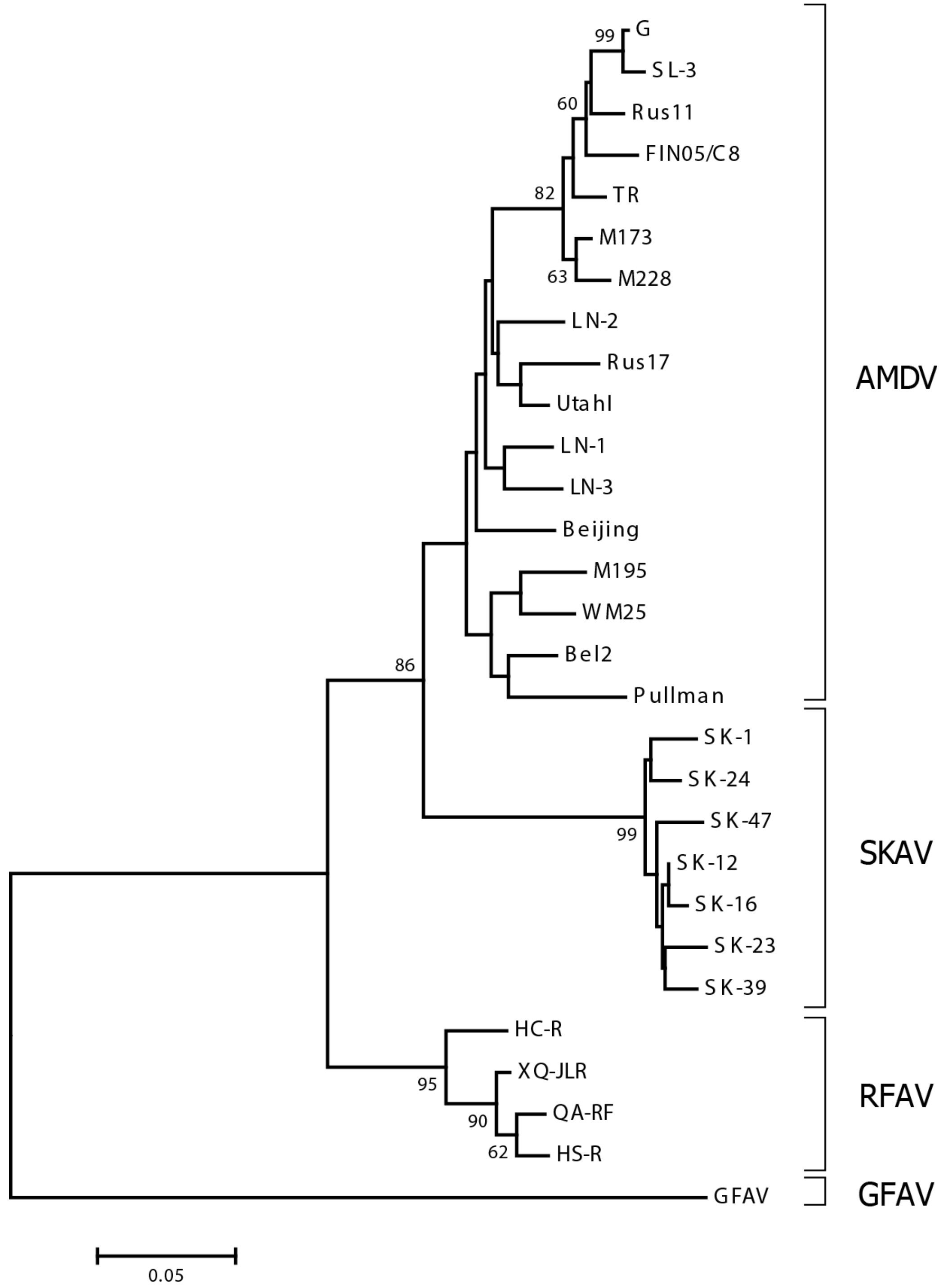
G
SL-3
Rus11
FIN05/C8
TR
M173
M228
LN-2
Rus17
Utahl
LN-1
LN-3
Beijing
M195
WM25
Bel2
Pullman
SK-1
SK-24
SK-47
SK-12
SK-16
SK-23
SK-39
HC-R
XQ-JLR
QA-RF
HS-R
GFAV
99
60
82
63
86
99
95
90
62
AMDV
SKAV
RFAV
GFAV
0.05
