# Supplementary material for: Full genetic characterization and epidemiology of a novel amdoparvovirus in striped skunk (Mephitis mephitis)
Source: Emerg Microbes Infect. 2017 May 10;6(5):e30–. doi: 10.1038/emi.2017.13 (PMC5520478; doi:10.1038/emi.2017.13)
Supplement: Supplementary Table S1 [file emi201713x3.pdf]

Supplemetary Table S1. Accession numbers and details of sequences used in this study

Strain	Genomic region*	Host	Year	Accession number	Figures	References
AMDV						
G	CG	Mink	Late 1970s	JN040434	2, 3, 5, S2	[1]
Utah I	CG	Mink	1963	Z18276	2, 3, 5, S2	[2]
SL-3	CG	Mink	Early 1980s	X97629	2, 3, 5, S2	[3]
LN-1	CG	Mink	2009	GU183265	2, 3, 5, S2	[4]
LN-2	CG	Mink	2009	GU183265	2, 3, 5, S2	[4]
LN-3	CG	Mink	2009	GU269892	2, 3, 5, S2	[4]
WM25	CG	Mink	2014	KT878961	2, 3, 5, S2	[5]
M195	CG	Mink	2014	KT878959	2, 3, 5, S2	[5]
M228	CG	Mink	2014	KT878960	2, 3, 5, S2	[5]
M173	CG	Mink	2014	KT878958	2, 3, 5, S2	[5]
Beijing	CG	Mink	2015	KT329428	2, 3, 5, S2	[6]
K	NS1	Mink	1982	X77084	2, 4, 5, S2	[7]
United	NS1	Mink	Not available	X77085	2, 5, S2	[8]
BCM-1	NS1	Mink	2015	KX981972	2, S2	This study
BCM-3	NS1	Mink	2015	KX981973	2, S2	This study
BCM-12	NS1	Mink	2015	KX981974	2, S2	This study
BCM-13	NS1	Mink	2015	KX981975	2, S2	This study
BCM-21	NS1	Mink	2015	KX981976	2, S2	This study
BCM-22	NS1	Mink	2015	KX981977	2, S2	This study
BCM-31	NS1	Mink	2015	KX981978	2, S2	This study
BCM-32	NS1	Mink	2015	KX981979	2, S2	This study
BCM-41	NS1	Mink	2015	KX981980	2, S2	This study
BCM-43	NS1	Mink	2015	KX981981	2, S2	This study
Pullman	VP2	Mink	1995	U39014	2, 5, S2	[9]
Rus17	VP2	Mink	Not available	KJ174164	2, 5, S2	Unpublished
TR	VP2	Mink	1990s	U39013	2, 5, S2	[9]
FIN05/C8	VP2	Mink	2005	GQ336866	2, 5, S2	[10]
Rus11	VP2	Mink	Not available	KJ174158	2, 5, S2	Unpublished
Bel2	VP2	Mink	Not available	KJ174161	2, 5, S2	Unpublished
SKAV						
SK-1	CG	Striped skunk	2011	KX981920	2, 3, 4, 5, S2	This study
SK-12	CG	Striped skunk	2014	KX981921	2, 3, 4, 5, S2	This study
SK-16	CG	Striped skunk	2014	KX981922	2, 3, 4, 5, S2	This study
SK-23	CG	Striped skunk	2014	KX981923	2, 3, 4, 5, S2	This study
SK-24	CG	Striped skunk	2014	KX981924	2, 3, 4, 5, S2	This study
SK-36	NS1	Striped skunk	2015	KX981971	2, 4, 5, S2	This study
SK-39	CG	Striped skunk	2015	KX981925	2, 3, 4, 5, S2	This study
SK-47	CG	Striped skunk	2015	KX981926	2, 3, 4, 5, S2	This study
SK-2	NS1	Striped skunk	2011	KX981927	4	This study
SK-3.1	NS1	Striped skunk	2011	KX981928	4	This study

SK-3.7	NS1	Striped skunk	2011	KX981929	4	This study
SK-4.1	NS1	Striped skunk	2012	KX981930	4	This study
SK-4.2	NS1	Striped skunk	2013	KX981931	4	This study
SK-4.3	NS1	Striped skunk	2013	KX981932	4	This study
SK-5	NS1	Striped skunk	2012	KX981933	4	This study
SK-6	NS1	Striped skunk	2013	KX981934	4	This study
SK-7.1	NS1	Striped skunk	2013	KX981935	4	This study
SK-7.3	NS1	Striped skunk	2013	KX981936	4	This study
SK-7.7	NS1	Striped skunk	2013	KX981937	4	This study
SK-9	NS1	Striped skunk	2013	KX981938	4	This study
SK-10	NS1	Striped skunk	2013	KX981939	4	This study
SK-11	NS1	Striped skunk	2013	KX981940	4	This study
SK-13	NS1	Striped skunk	2013	KX981941	4	This study
SK-15.1	NS1	Striped skunk	2014	KX981942	4	This study
SK-15.2	NS1	Striped skunk	2014	KX981943	4	This study
SK-17	NS1	Striped skunk	2014	KX981944	4	This study
SK-18	NS1	Striped skunk	2014	KX981945	4	This study
SK-19	NS1	Striped skunk	2014	KX981946	4	This study
SK-20	NS1	Striped skunk	2014	KX981947	4	This study
SK-21.1	NS1	Striped skunk	2014	KX981948	4	This study
SK-21.3	NS1	Striped skunk	2014	KX981949	4	This study
SK-22	NS1	Striped skunk	2014	KX981950	4	This study
SK-25	NS1	Striped skunk	2014	KX981951	4	This study
SK-26	NS1	Striped skunk	2014	KX981952	4	This study
SK-27.6	NS1	Striped skunk	2014	KX981953	4	This study
SK-27.9	NS1	Striped skunk	2014	KX981954	4	This study
SK-28	NS1	Striped skunk	2014	KX981955	4	This study
SK-29	NS1	Striped skunk	2014	KX981956	4	This study
SK-31	NS1	Striped skunk	2014	KX981957	4	This study
SK-32	NS1	Striped skunk	2014	KX981958	4	This study
SK-33	NS1	Striped skunk	2014	KX981959	4	This study
SK-35	NS1	Striped skunk	2015	KX981960	4	This study
SK-37	NS1	Striped skunk	2015	KX981961	4	This study
SK-38	NS1	Striped skunk	2015	KX981962	4	This study
SK-40	NS1	Striped skunk	2015	KX981963	4	This study
SK-41	NS1	Striped skunk	2015	KX981964	4	This study
SK-42	NS1	Striped skunk	2015	KX981965	4	This study
SK-43	NS1	Striped skunk	2015	KX981966	4	This study
SK-44	NS1	Striped skunk	2015	KX981967	4	This study
SK-45	NS1	Striped skunk	2015	KX981968	4	This study
SK-46	NS1	Striped skunk	2015	KX981969	4	This study
SK-48	NS1	Striped skunk	2015	KX981970	4	This study
S2	VP2	Striped skunk	2008	HM623397	5	[11]
S4	VP2	Striped skunk	2008	HM623405	5	[11]
S4	NS1	Striped skunk	2008	HM623354	5	[11]
S5	NS1	Striped skunk	2008	HM623355	5	[11]
S5	VP2	Striped skunk	2008	HM623406	5	[11]
S14	VP2	Striped skunk	2008	HM623393	5	[11]
S14	NS1	Striped skunk	2008	HM623345	5	[11]

S19	VP2	Striped skunk	2008	HM623394	5	[11]
S19	NS1	Striped skunk	2008	HM623346	5	[11]
S22	VP2	Striped skunk	2008	HM623395	5	[11]
S22	NS1	Striped skunk	2008	HM623347	5	[11]
S29	VP2	Striped skunk	2008	HM623396	5	[11]
S29	NS1	Striped skunk	2008	HM623348	5	[11]
S32	VP2	Striped skunk	2008	HM623398	5	[11]
S36	VP2	Striped skunk	2008	HM623399	5	[11]
S36	NS1	Striped skunk	2008	HM623349	5	[11]
S37	VP2	Striped skunk	2008	HM623400	5	[11]
S37	NS1	Striped skunk	2008	HM623350	5	[11]
S38	VP2	Striped skunk	2008	HM623401	5	[11]
S38	NS1	Striped skunk	2008	HM623351	5	[11]
S40	VP2	Striped skunk	2008	HM623402	5	[11]
S40	NS1	Striped skunk	2008	HM623352	5	[11]
S41	VP2	Striped skunk	2008	HM623403	5	[11]
S43	BP2	Striped skunk	2008	HM623404	5	[11]
S43	NS1	Striped skunk	2008	HM623353	5	[11]
California	VP2	Striped skunk	2006	Not available	5	[12]
M8	VP2	Mink	2005	HM623384	5	[13]
M8	NS1	Mink	2005	HM623339	5	[13]
ON09-02-8703	NS1	Mink	2009	KT878930	5	[5]
**RFAV**						
HC-R	CG	Raccoon dog	2012	KJ396348	2, 3, S2	[14]
XQ-JLR	CG	Raccoon dog	2013	KJ396350	2, 3, S2	[14]
QA-RF	CG	Raccoon dog, Arctic fox	2012	KJ396349	2, 3, S2	[14]
HS-R	CG	Raccoon dog	2012	KJ396347	2, 3, S2	[14]
**GFAV**						
GFAV	CG	Gray fox	2009	JN202450	2, 3, S2	[15]
**RFFAV**						
S40	NS1	Red fox	2013	KF823809	/	[16]
S40	VP2	Red fox	2013	KF823808	/	[16]

*CG: complete genome.

References

1 Huang Q, Deng X, Best SM, Bloom ME, Li Y, Qiu J. Internal polyadenylation of parvoviral precursor mRNA limits progeny virus production. *Virology* 2012; **426**: 167–177.

2 Bloom ME, Alexandersen S, Perryman S, Lechner D, Wolfinbarger JB. Nucleotide sequence and genomic organization of Aleutian mink disease parvovirus (ADV): sequence comparisons between a nonpathogenic and a pathogenic strain of ADV. *J Virol* 1988; **62**: 2903–2915.

3 Schuierer S, Bloom ME, Kaaden OR, Truyen U. Sequence analysis of the lymphotropic Aleutian disease parvovirus ADV-SL3. *Arch Virol* 1997; **142**: 157–166.

4 Li Y, Huang J, Jia Y, Du Y, Jiang P, Zhang R. Genetic characterization of Aleutian mink disease viruses isolated in China. *Virus Genes* 2012; **45**: 24–30.

5 Canuti M, O'Leary KE, Hunter BD, Spearman G, Ojkic D, Whitney HG *et al.* Driving forces behind the evolution of the Aleutian mink disease parvovirus in the context of intensive farming. *Virus Evol* 2016; **2**: vew004.

6 Xi J, Wang J, Yu Y, Zhang X, Mao Y, Hou Q *et al.* Genetic characterization of the complete genome of an Aleutian mink disease virus isolated in north China. *Virus Genes* 2016; **52**: 463–473.

7 Gottschalck E, Alexandersen S, Cohn A, Poulsen LA, Bloom ME, Aasted B. Nucleotide sequence analysis of Aleutian mink disease parvovirus shows that multiple virus types are present in infected mink. *J Virol* 1991; **65**: 4378–4386.

8 Gottschalck E, Alexandersen S, Storgaard T, Bloom ME, Aasted B. Sequence comparison of the non-structural genes of four different types of Aleutian mink disease parvovirus indicates an unusual degree of variability. *Arch Virol* 1994; **138**: 213–231.

9 Oie KL, Durrant G, Wolfinbarger JB, Martin D, Costello F, Perryman S *et al.* The relationship between capsid protein (VP2) sequence and pathogenicity of Aleutian mink disease parvovirus (ADV): a possible role for raccoons in the transmission of ADV infections. *J Virol* 1996; **70**: 852–861.

10 Knuuttila A, Uzcátegui N, Kankkonen J, Vapalahti O, Kinnunen P. Molecular epidemiology of Aleutian mink disease virus in Finland. *Vet Microbiol* 2009; **133**: 229–238.

11 Nituch LA, Bowman J, Wilson PJ, Schulte-Hostedde AI. Aleutian mink disease virus in striped skunks (Mephitis mephitis): evidence for cross-species spillover. *J Wildl Dis* 2015; **51**: 389–400.

12 Allender MC, Schumacher J, Thomas KV, McCain SL, Ramsay EC, James EW *et al.* Infection with Aleutian disease virus-like virus in a captive striped skunk. *J Am Vet Med Assoc* 2008; **232**: 742–746.

13 Nituch LA, Bowman J, Wilson P, Schulte-Hostedde AI. Molecular epidemiology of Aleutian disease virus in free-ranging domestic, hybrid, and wild mink. *Evol Appl* 2012; **5**: 330–340.

14 Shao X-Q, Wen Y-J, Ba H-X, Zhang X-T, Yue Z-G, Wang K-J *et al.* Novel amdoparvovirus infecting farmed raccoon dogs and Arctic foxes. *Emerg Infect Dis* 2014; **20**: 2085–2088.

15 Li L, Pesavento PA, Woods L, Clifford DL, Luff J, Wang C *et al.* Novel amdovirus in gray foxes. *Emerg Infect Dis* 2011; **17**: 1876–1878.

16 Bodewes R, Ruiz-Gonzalez A, Schapendonk CME, van den Brand JMA, Osterhaus ADME, Smits SL. Viral metagenomic analysis of feces of wild small carnivores. *Virol J* 2014; **11**: 89.
