# Supplementary material for: Full genetic characterization and epidemiology of a novel amdoparvovirus in striped skunk (Mephitis mephitis)
Source: Emerg Microbes Infect. 2017 May 10;6(5):e30–. doi: 10.1038/emi.2017.13 (PMC5520478; doi:10.1038/emi.2017.13)
Supplement: Supplementary Table S2 [file emi201713x4.pdf]

**Supplementary Table S2. Splicing profile and viral protein details of AMDV and SKAV strains sequenced in this study and compared to the reference sequence AMDV-G**

Splicing sites were determined following what was experimentally demonstrated. Scores were assigned to donor and acceptor sites using the splicing prediction algorithm NNSPLICE from fruitfly.org.

| Strain | Protein | Size (aa) | Exon position* | Donor score$ | Exon position*,$ | Acceptor score$ |
|---|---|---|---|---|---|---|
| AMDV | | | | | | |
| G | NS1 | 641 | 206-1961 | 1.00 | 2042-2211 | 0.94 |
| | NS2 | 114 | 206-384 | 0.95 | 2042-2207 | 0.94 |
| | NS3 | 87 | 206-384 | 0.95 | 1737-1821 | / |
| | VP1 | 681 | 2204-2213 | 0.84 | 2287-4322 | 0.95 |
| | VP2 | 638 | 2406-4322 | / | / | / |
| BCM-1 | NS1 | 641 | 69-1824 | 1.00 | 1906-2075 | 0.93 |
| | NS2 | 114 | 69-247 | 0.93 | 1906-2071 | 0.93 |
| | NS3 | 92 | 69-247 | 0.93 | 1600-1699 | / |
| BCM-3 | NS1 | 641 | 69-1824 | 1.00 | 1906-2075 | 0.93 |
| | NS2 | 114 | 69-247 | 0.93 | 1906-2071 | 0.93 |
| | NS3 | 92 | 69-247 | 0.93 | 1600-1699 | / |
| BCM-12 | NS1 | 641 | 44-1799 | 1.00 | 1880-2049 | 0.95 |
| | NS2 | 114 | 44-222 | 0.94 | 1880-2045 | 0.95 |
| | NS3 | 87 | 44-222 | 0.94 | 1575-1659 | / |
| BCM-13 | NS1 | 641 | 45-1800 | 1.00 | 1881-2050 | 0.93 |
| | NS2 | 114 | 45-223 | 0.90 | 1881-2046 | 0.93 |
| | NS3 | 92 | 45-223 | 0.90 | 1576-1675 | / |
| BCM-21 | NS1 | 641 | 69-1824 | 1.00 | 1905-2074 | 0.95 |
| | NS2 | 114 | 69-247 | 0.93 | 1905-2070 | 0.95 |
| | NS3 | 87 | 69-247 | 0.93 | 1600-1684 | / |
| BCM-22 | NS1 | 641 | 69-1824 | 1.00 | 1906-2075 | 0.93 |
| | NS2 | 114 | 69-247 | 0.93 | 1906-2071 | 0.93 |
| | NS3 | 92 | 69-247 | 0.93 | 1600-1699 | / |
| BCM-31 | NS1 | 641 | 2-1757 | 1.00 | 1838-2007 | 0.93 |
| | NS2 | 114 | 2-180 | 0.93 | 1838-2003 | 0.93 |
| | NS3 | 92 | 2-180 | 0.93 | 1533-1632 | / |
| BCM-32 | NS1 | 641 | 69-1824 | 1.00 | 1905-2074 | 0.94 |
| | NS2 | 114 | 69-247 | 0.94 | 1905-2070 | 0.94 |
| | NS3 | 87 | 69-247 | 0.94 | 1600-1684 | / |
| BCM-41 | NS1 | 641 | 69-1824 | 1.00 | 1905-2074 | 0.93 |
| | NS2 | 114 | 69-247 | 0.93 | 1905-2070 | 0.93 |
| | NS3 | 92 | 69-247 | 0.93 | 1600-1699 | / |
| BCM-43 | NS1 | 641 | 51-1806 | 1.00 | 1887-2056 | 0.95 |
| | NS2 | 114 | 51-229 | 0.95 | 1887-2052 | 0.95 |
| | NS3 | 87 | 51-229 | 0.95 | 1582-1666 | / |

| | | | | | | |
|---|---|---|---|---|---|---|
| SKAV | | | | | | |
| SK-1 | NS1 | 641 | 69-1824 | 1.00 | 1907-2076 | 0.93 |
| | NS2 | 114 | 69-247 | 0.94 | 1907-2072 | 0.93 |
| | NS3 | 71 | 69-247 | 0.94 | 1600-1636 | / |
| | VP1 | 677 | 2069-2078 | 0.99 | 2152-4175 | 0.95 |
| | VP2 | 634 | 2271-4175 | / | / | / |
| SK-12 | NS1 | 641 | 68-1823 | 1.00 | 1906-2075 | 0.94 |
| | NS2 | 114 | 68-246 | 0.94 | 1906-2071 | 0.94 |
| | NS3 | 71 | 68-246 | 0.94 | 1599-1635 | / |
| | VP1 | 677 | 2068-2077 | 0.99 | 2151-4174 | 0.88 |
| | VP2 | 634 | 2270-4174 | / | / | / |
| SK-16 | NS1 | 641 | 69-1824 | 1.00 | 1907-2076 | 0.94 |
| | NS2 | 114 | 69-247 | 0.94 | 1907-2072 | 0.94 |
| | NS3 | 71 | 69-247 | 0.94 | 1600-1636 | / |
| | VP1 | 677 | 2069-2078 | 0.99 | 2152-4175 | 0.90 |
| | VP2 | 634 | 2271-4175 | / | / | / |
| SK-23 | NS1 | 641 | 69-1824 | 1.00 | 1907-2076 | 0.94 |
| | NS2 | 114 | 69-247 | 0.94 | 1907-2072 | 0.94 |
| | NS3 | 71 | 69-247 | 0.94 | 1600-1636 | / |
| | VP1 | 677 | 2069-2078 | 0.99 | 2152-4175 | 0.95 |
| | VP2 | 634 | 2271-4175 | / | / | / |
| SK-24 | NS1 | 641 | 69-1824 | 1.00 | 1907-2076 | 0.97 |
| | NS2 | 114 | 69-247 | 0.94 | 1907-2072 | 0.97 |
| | NS3 | 71 | 69-247 | 0.94 | 1600-1636 | / |
| | VP1 | 677 | 2069-2078 | 0.99 | 2152-4175 | 0.90 |
| | VP2 | 634 | 2271-4175 | / | / | / |
| SK-36 | NS1 | 641 | 69-1824 | 1.00 | 1907-2076 | 0.94 |
| | NS2 | 114 | 69-247 | 0.94 | 1907-2072 | 0.94 |
| | NS3 | 71 | 69-247 | 0.94 | 1600-1636 | / |
| SK-39 | NS1 | 641 | 69-1824 | 1.00 | 1907-2076 | 0.94 |
| | NS2 | 114 | 69-247 | 0.94 | 1907-2072 | 0.94 |
| | NS3 | 71 | 69-247 | 0.94 | 1600-1636 | / |
| | VP1 | 677 | 2069-2078 | 0.99 | 2152-4175 | 0.9 |
| | VP2 | 634 | 2271-4175 | / | / | / |
| SK-47 | NS1 | 641 | 69-1824 | 1.00 | 1907-2076 | 0.93 |
| | NS2 | 114 | 69-247 | 0.94 | 1907-2072 | 0.93 |
| | NS3 | 71 | 69-247 | 0.94 | 1600-1636 | / |
| | VP1 | 677 | 2069-2078 | 0.99 | 2152-4175 | 0.90 |
| | VP2 | 634 | 2271-4175 | / | / | / |

* Positions refer to each sequence as published in GenBank with accession numbers listed in Supplementary Table S1.

[$] A / indicates that the site was absent (for VP2) or not detected using the splicing prediction algorithm (for NS3).
