# Supplementary material for: Full genetic characterization and epidemiology of a novel amdoparvovirus in striped skunk (Mephitis mephitis)
Source: Emerg Microbes Infect. 2017 May 10;6(5):e30–. doi: 10.1038/emi.2017.13 (PMC5520478; doi:10.1038/emi.2017.13)
Supplement: Supplementary Table S3 [file emi201713x5.pdf]

Supplementary Table S3. Pairwise percentage sequence identities (1 – p-distance) within and between different SKAV lineages and sub-lineages

	Average	Range
**Within clade**		
1	98.2	95.9-100
2	97	92.5-100
2A	97.9	95.6-100
2B	93.9	93.4-94.6
**Between clades**		
1 vs. 2	91.4	89.6-93.3
2A vs. 1	91.5	89.7-93.3
2B vs. 1	90.9	89.6-92.4
2A vs. 2B	94.1	92.5-95.2
